# The Platelet-Activating Factor Receptor’s Association with the Outcome of Ovarian Cancer Patients and Its Experimental Inhibition by Rupatadine

**DOI:** 10.3390/cells10092337

**Published:** 2021-09-07

**Authors:** Eileen Deuster, Ivi Hysenaj, Maja Kahaly, Elisa Schmoeckel, Doris Mayr, Susanne Beyer, Thomas Kolben, Anna Hester, Fabian Kraus, Anca Chelariu-Raicu, Alexander Burges, Sven Mahner, Udo Jeschke, Fabian Trillsch, Bastian Czogalla

**Affiliations:** 1 Department of Obstetrics and Gynecology, University Hospital, Ludwig Maximilians University (LMU) Munich, 81377 Munich, Germany; eileen.deuster@kgu.de (E.D.); Ivi.hysenaj@med.uni-muenchen.de (I.H.); maja.kahaly@med.uni-muenchen.de (M.K.); Susanne.Beyer@med.uni-muenchen.de (S.B.); Thomas.Kolben@med.uni-muenchen.de (T.K.); Anna.Hester@med.uni-muenchen.de (A.H.); Fabian.Kraus@med.uni-muenchen.de (F.K.); Anca.Chelariu-Raicu@med.uni-muenchen.de (A.C.-R.); Alexander.Burges@med.uni-muenchen.de (A.B.); Sven.Mahner@med.uni-muenchen.de (S.M.); Udo.Jeschke@med.uni-muenchen.de (U.J.); Fabian.Trillsch@med.uni-muenchen.de (F.T.); 2 Institute of Pathology, Faculty of Medicine, Ludwig Maximilians University (LMU) Munich, 81377 Munich, Germany; elisa.schmoeckel@med.uni-muenchen.de (E.S.); Doris.Mayr@med.uni-muenchen.de (D.M.); 3 Department of Obstetrics and Gynecology, University Hospital Augsburg, 86156 Augsburg, Germany

**Keywords:** ovarian cancer, platelet-activating factor receptor (PAFR), rupatadine, platelet-activating factor (PAF)

## Abstract

The platelet-activating factor receptor (PAFR) and its ligand (PAF) are important inflammatory mediators that are overexpressed in ovarian cancer. The receptor is an important player in ovarian cancer development. In this study, we aimed to evaluate the prognostic value of PAFR in epithelial ovarian cancer (EOC) and the potential use of its antagonist, rupatadine, as an experimental treatment. Tissue microarrays of ovarian cancer patients, most markedly those with a non-mucinous subtype, immunohistochemically overexpressed PAFR. Elevated cytoplasmic PAFR expression was found to significantly and independently impair patients’ overall and recurrence-free survival (OS: median 83.48 vs. 155.03 months; *p* = 0.022; RFS: median 164.46 vs. 78.03 months; *p* = 0.015). In vitro, the serous ovarian cancer subtypes especially displayed an elevated PAFR gene and protein expression. siRNA knockdown of PAFR decreased cell proliferation significantly, thus confirming the receptor’s protumorigenic effect on ovarian cancer cells. The clinically approved PAFR antagonist rupatadine effectively inhibited in vitro cell proliferation and migration of ovarian cancer cells. PAFR is a prognostic marker in ovarian cancer patients and its inhibition through rupatadine may have important therapeutic implications in the therapy of ovarian cancer patients.

## 1. Introduction

Ovarian cancer is one of the five leading types of cancer death among women of all ages [1]. Over recent years, the outcome of ovarian cancer patients has remained poor, with a five-year survival rate of less than 45% [2]. The high case fatality rate of ovarian cancer is mostly attributed to late-stage diagnosis due to an occult growth of the tumor within the peritoneal cavity and a lack of screening methods. Therapeutic options are still limited. Debulking surgery is the most effective curative treatment choice. Additionally, adjuvant platinum-based chemotherapy combined with anti-angiogenic agents or followed by poly-ADP-ribose-polymerase inhibitors are established treatment options [3]. However, most ovarian cancer patients are diagnosed when metastasis has already occurred [4]. Hence, it is vital to develop new and effective prognostic and therapeutic options.

Chronic inflammation is a key factor in the pathogenesis of ovarian cancer and other malignancies [5,6,7]. In ovarian cancer, chronic inflammation in the tumor environment can be linked to tumor formation, progress, and metastasis. Important inflammatory lipid mediators are the platelet-activating factor (PAF) and its receptor (PAFR). PAFR is a G-protein-coupled receptor that signals through G-proteins and associated phosphorylation pathways. The receptors’ sole ligand, PAF, which was first described as an inducer of platelet degranulation and aggregation, is an essential proinflammatory activator of neutrophils, macrophages, platelets, lymphocytes, and endothelial cells [8].

The role of PAF and PAFR in various cancers, including ovarian cancer, has been investigated in recent years. In ovarian cancer, PAFR is overexpressed and has been identified as an important player in tumor development, metastasis, anti-apoptosis, and angiogenesis [9,10,11,12,13]. However, the receptor’s significance for long-term survival of ovarian cancer patients is not yet known. The inhibition of PAFR with specific antagonists (WEB 2086 and Ginkgolide B) showed promising antiproliferative effects with reduced tumor growth in ovarian cancer models [14,15]. Another inhibitor of PAFR is rupatadine. which has not yet been evaluated in ovarian cancer [16]. It is a clinically approved and used antihistaminic drug for allergic diseases [17]. Due to its inhibition of PAFR and good safety profile, we considered it as a potential drug candidate in ovarian cancer [16].

The study aimed to first assess the clinical importance of PAFR on long-term patients’ outcomes. On a molecular level, we examined PAFR gene and protein expression in different subtypes of ovarian cancer cells. To investigate the role of PAFR in epithelial ovarian cancer (EOC), we performed PAFR gene knockdown and evaluated its effect on EOC proliferation. In a drug repurposing approach, we antagonized PAFR with the clinically approved rupatadine. To evaluate the antagonists’ influence on EOC development, we conducted proliferation and migration assays.

## 2. Materials and Methods

### 2.1. Patients

Ovarian cancer samples from 156 patients who underwent surgery for EOC from 1990 to 2002 at the Department of Obstetrics and Gynecology, Ludwig Maximilian University in Munich, Germany were included (Table 1). Written informed consent was obtained from all patients. We only included patients with a definite diagnosis of ovarian cancer in this study; borderline tumors or benign tumors were excluded. Clinical data were retrieved from the patients’ charts, and the Munich Cancer Registry (MCR) provided the follow-up data. The histological classification (serous (*n* = 110), endometrioid (*n* = 21), mucinous (*n* = 13), clear cell (*n* = 12)) and tumor grading according to the WHO criteria were performed by a gynecological pathologist. Unfortunately, the BRCA status of the patient collective was not accessible. Most patients (67.9%) presented with advanced stage disease (FIGO III and FIGO IV). All patients that were staged as FIGO II–IV received carboplatin and paclitaxel as adjuvant chemotherapy.

### 2.2. Ethics Approval

The Ethics Committee of the Ludwig Maximilian University, Munich, Germany, approved this study (approval numbers 227-09 and 18-392). All tissue samples utilized for this investigation were collected from material from the archives of the Department of Obstetrics and Gynecology, University Hospital, LMU Munich, Munich, Germany, having initially been used for pathological diagnostics. The diagnostic procedures were concluded before the current study was conducted. During the analysis, the observers were fully blinded for patients’ data.

### 2.3. Immunohistochemistry

Tissue microarrays of formalin-fixed paraffin-embedded ovarian cancer tissue samples were prepared and stained as previously described [18,19]. As primary antibody we used the polyclonal rabbit IgG anti-PAFR antibody (Abcam, Cambridge, UK). Kidney tissues served as a positive and negative control.

The intensity of the PAFR expression was assessed by the immunoreactive score (IRS). This is a semi-quantitative scoring system which is well established. The IRS is obtained by multiplying the staining intensity (negative = 0, weak = 1, moderate = 2, strong = 3) with the percentage of positive cells (negative = 0, ≤10% = 1, ≥11%, ≤50% = 2, ≥51%, ≤80% = 3, ≥81% = 4) resulting in an IR score between 0 and 12. In the cytoplasm, a score between 0 and 3 was marked as low, and between 4 and 12 as high.

In our study, two independent scorers analyzed the intensity and distribution pattern of PAFR in the cytoplasm (Table S3). The light microscope “Immunohistochemistry Type 307–148.001 512 686” by Leitz (Wetzlar, Germany) was used. The camera was produced by Fissler (IH-Camera 3CCD Colour Video Camera). For image acquisition, the software “Discuss Version 4,602,017-#233 (Carl C. Hilgers Technical Office) was used. Image bit depth: 24 mm; time and space resolution data: 760 + 574 pixels.

### 2.4. Cells, Culture Conditions, and Reagents

The OVCAR-3 (serous, BRCA WT), UWB1.289 (serous, BRCA1 negative), ES-2 (clear cell), and TOV 112D (endometrioid) cell lines were obtained from the American Tissue Culture Collection (ATCC, Rockville, MD, USA). All cell lines were cultured in RPMI 1640 medium (ThermoFisher Scientific, Waltham, MA, USA) containing 10% fetal bovine serum (FBS) without antimycotics/antibiotics in a humidified atmosphere (37 °C, 5% CO_2_). All cell lines were tested for mycoplasma before the experiments.

### 2.5. RT-qPCR

RNA isolation was performed according to the manufacturer’s protocol by using the RNeasy Mini Kit (Qiagen, Venlo, The Netherlands). RNA concentrations were adjusted, and cDNA synthesis was carried out with the MMLV Reverse Transcriptase 1st-Strand cDNA Synthesis Kit (Epicentre, Madison, WI, USA). qPCR was carried out with FastStart Essential DNA Probes Master and gene-specific primers (Roche, Basel, Switzerland). The primer sequences are available in Table S1. The relative expression was calculated by the 2-ΔΔCt method, applying β-Actin and GAPDH as housekeeping genes [20].

### 2.6. siRNA Knockdown

For siRNA knockdown experiments, UWB1.289 cells were transfected with small interfering RNA (siRNA; 3 different sequences for PAFR are available in Table S2) using Lipofectamine RNAiMAX reagent (Invitrogen, Carlsbad, CA, USA). A scrambled negative control siRNA (Qiagen, Hilden, Germany) was used as a reference. UWB1.289 cells were cultured in six-well plates and transfected when cell density reached 60–70%. Opti-MEM Reduced Serum Medium (Thermo Fisher Scientific, Waltham, MA, USA) containing siRNA-PAFR and Lipofectamine RNAiMAX was used for the treatment of the cells. After 36 h, cells were harvested and used for further analysis.

### 2.7. Western Blotting

Wells were washed twice in ice-cold PBS and lysed for 15 min at 4 °C in 200 µL RIPA buffer containing protease inhibitor (Sigma-Aldrich Co., St. Louis, MO, USA). The protein concentration of the lysates was determined by Bradford assay. Then, cellular extracts were subjected to sodium dodecyl sulfate-polyacrylamide gel and transferred onto a polyvinylidene fluoride membrane (EMD Millipore, Billerica, MA, USA). After blocking the membrane for 1 h with casein (Vector Laboratories, Burlingame, CA, USA), blots were incubated with diluted primary antibodies gently shaking overnight at 4 °C. As primary antibodies, a rabbit polyclonal antibody against PAFR (1:200 dilution; Cayman, Ann Arbor, MI, USA) and a mouse monoclonal antibody against β-actin (1:1000 dilution; Sigma, St. Louis, MO, USA) were employed. β-actin Western blots served as controls. Subsequently, blots were washed with 1:10 casein three times and then subjected to biotinylated anti-mouse/anti-rabbit IgG secondary antibodies and ABC-AmP reagent (VECTASTAIN ABC-AmP Kit for rabbit IgG; Vector Laboratories, Burlingame, CA, USA). The antibody complexes were visualized with 5-bromo-4-chloro-3-indolylphosphate/nitroblue tetrazolium chromogenic substrate (Vectastain ABC-AmP Kit; Vector Laboratories, Burlingame, CA, USA). Western blotting detection and analysis was performed with Bio-Rad Universal Hood II and the corresponding software Quantity One (Bio-Rad Laboratories Inc., Hercules, CA, USA).

### 2.8. Cell Proliferation

Cell proliferation of OVCAR-3, UWB1.289, ES-2, and TOV 112D was assessed with a dimethyl-thiazolyl-diphenyl-tetrazolium bromide (MTT) assay (Roche, Mannheim, Germany). Cells were seeded at a density of 5 × 10^3^ cells per well into 96-well plates and incubated in RPMI 1640 medium with 10% FBS for 24 h before being treated. The siRNA transfection (PAFR gene knockdown) was conducted as described above. The cells were inhibited with the PAFR antagonist rupatadine (Sigma-Aldrich Co., St. Louis, MO, USA, catalog number CDS022916) or the respective carrier solution (ethanol, DMSO) at the indicated concentrations. After treatment for 72 h, MTT-labelling (5 mg/mL in PBS dye solution) was performed according to the manufacturer’s manual. A spectrophotometric reading analyzed absorbance with the Microplate Reader (BioTek, Winooski, VT, USA) at 595 nm.

### 2.9. Wound Healing

The cells were seeded in 12-well plates (4 × 10^5^) for 24 h. To create an artificial wound, the central fields of confluent monolayers were scratched manually with 200 μL pipette tips through the entire center of the wells. Digital images were taken after treatment with rupatadine at 0 h and 24 h. Cell migration was monitored with an inverse phase-contrast microscope (Leica Dmi1, Wetzlar, Germany). Photos of cell migration area were analyzed with the software Image J (https://imagej.nih.gov/ij/, last accessed on the 10 October 2017), and the cell migration area was calculated as the difference of the scratch areas between 24 h and 0 h.

### 2.10. Statistical Analysis

Data were analyzed using SPSS (v21, IBM, Armonk, New York, NY, USA) and GraphPad Prism Version 8.00 software (San Diego, CA, USA). Spearman coefficient was employed to correlate data. Histological subtypes were encoded in the following way: 1 = serous, 2 = clear cell, 3 = endometroid, and 4 = mucinous.

Kaplan–Meier curves were drawn to compare survival times between groups. The chi-square statistic of the log-rank (Mantel–Cox) test was applied to test differences in overall and recurrence-free survival for significance. Cut-off points were obtained through the receiver operator curve (ROC) [21,22,23]. Multivariate Cox-regression was performed to analyze covariates. For unpaired data, if D’Agostino–Pearson omnibus normality test indicated Gaussian distribution, an unpaired *t*-test for side-by-side comparisons or one-way ANOVA with Tukey’s post-test for multiple comparisons was performed. Statistical significance for all tests was set as *p* = 0.05 and data was expressed as means ± SD.

## 3. Results

### 3.1. Elevated Cytoplasmic PAFR Expression in Serous, Clear Cell, and Endometrioid Ovarian Cancer Patients

**Figure 1:**
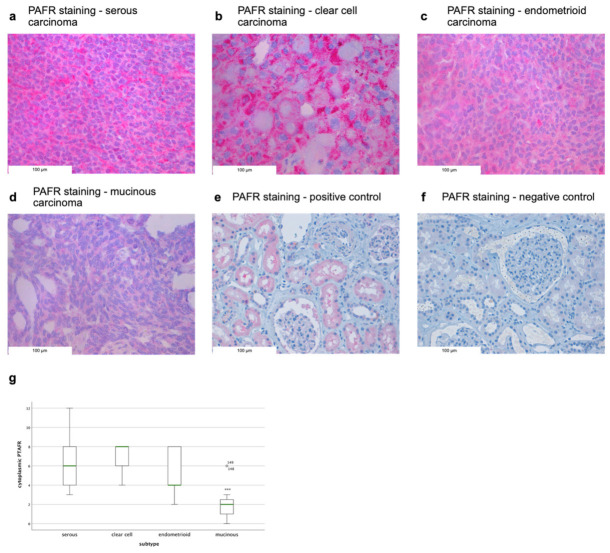
PAFR expression varies in different ovarian cancer subtypes. (**a**) Raised cytoplasmic PAFR staining in serous, (**b**) clear cell, and (**c**) endometrioid cancer cells compared to the (**d**) mucinous subtype. (**e**) PAFR positive and (**f**) PAFR negative control in the kidney. (**g**) The mucinous subtype demonstrates a significantly lower cytoplasmic PAFR staining expression compared to the other histological subtypes (*p* = 0.000) as analyzed by one-way ANOVA test and shown as *** *p* ≤ 0.001. Two patient outliers are indicated (148, 149).

Out of 148 successfully stained ovarian cancer specimens, 147 (99%) samples showed a positive cytoplasmic PAFR expression (Table S3). In the cytoplasm, the median immunoreactive score (IRS) was 6 (0–12). When analyzing the staining distribution between the different histological subtypes, the serous (median IRS = 6), clear cell (median IRS = 8), and endometrioid (median IRS = 4) samples demonstrate a significant elevated expression compared to the mucinous subtype (median IRS = 2; *p* = 0.000, Figure 1).

### 3.2. PAFR Expression Correlates with Clinical and Pathological Data

The correlation between cytoplasmic PAFR expression and clinical and pathological data such as histology, FIGO, and grading was analyzed. A significant correlation was found between cytoplasmic PAFR expression and histology (*p* = 0.011, Rho = −0.222). This means that there are significant differences between the histological subtypes. High grading in serous cancer patients correlated significantly with cytoplasmic PAFR expression (*p* = 0.031, Rho = 0.189, Table 2).

### 3.3. PAFR as a Negative Independent Prognostic Factor in Ovarian Cancer Patients

**Figure 2:**
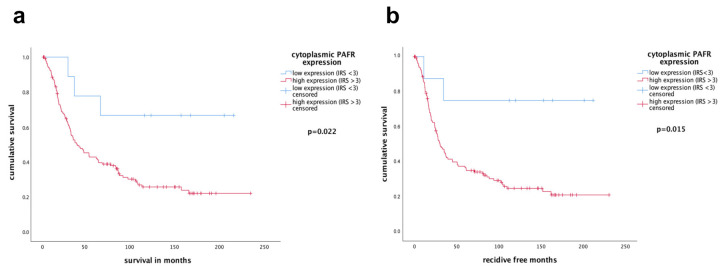
PAFR expression is a prognostic factor in ovarian cancer patients. (**a**) Overall and (**b**) recurrence-free survival of patients whose tumors expressed cytoplasmic PAFR at high levels was compared to those with low PAFR expression by the log rank test. Kaplan–Meier survival plots were drawn. Ovarian cancer patients with elevated PAFR expression in the cytoplasm survived significantly shorter (**a**) and demonstrated a shorter recurrence-free survival time (**b**) compared to the group of patients with low PAFR expression.

In this study, patients’ median survival time was 51.2 ± 57.6 months, and median months free of recurrence were 56.4 ± 57.6. The median age of the women were 62 ± 12 years with range of 31–88 years. Ovarian cancer patients with an increased cytoplasmic PAFR expression (IRS > 3) had a significantly worse outcome compared to the other subgroup. The mean survival time of patients with an elevated PAFR expression (IRS > 3) was 83.48 months vs. 155.03 months for patients with low expression (IRS < 3) (*p* = 0.022, Figure 2a). Similarly, patients with raised PAFR expression had a significantly higher chance of cancer recurrence (*p* = 0.015 164.46 vs. 78.03 months, Figure 2b).

In a multivariate Cox-regression analysis, we analyzed various clinicopathological parameters, as well as cytoplasmic PAFR expression, for recurrence-free survival. Patients with elevated cytoplasmic PAFR expression demonstrated a higher hazard ratio (HR = 4.069) than those with lower PAFR expression (*p* = 0.045). Out of the other coefficients, FIGO status was proven to significantly increase the hazard of patients (HR = 4.316; *p* = 0.000, Table 3).

### 3.4. PAFR Knockdown Reduced Ovarian Cancer Cell Proliferation

We first detected PAFR gene and protein expression in different ovarian cancer cell lines. In the PCR, the expression of PAFR was significantly higher in the serous, BRCA-1 negative ovarian cancer cell lines (UWB1.289) compared to the other groups (*p* ≤ 0.0001, Figure S1). We further analyzed PAFR protein expression by Western blot. The PAFR protein band was highly expressed in the UWB1.289 cell line. TOV 112D, OVCAR-3, and ES-2 also showed protein expression. This data is consistent with previous findings that PAFR is overexpressed in malignant ovarian cancer [24].

**Figure 3:**
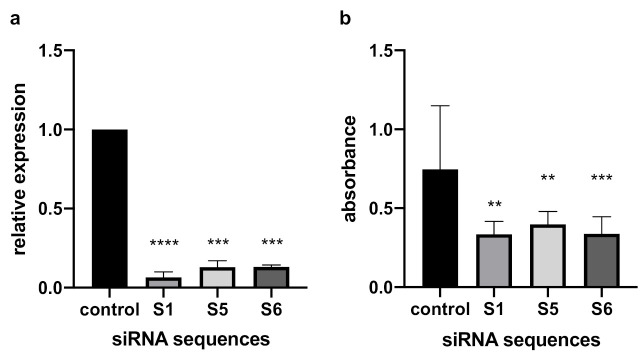
PAFR siRNA knockdown reduces proliferation of UWB1.289 ovarian cancer cells. (**a**) The upregulation of PAFR gene expression was significantly decreased by the three different siRNA sequences used, as shown by RT-qPCR. (**b**) Compared to the UWB1.289 cells with increased PAFR expression, the PAFR knockdown cancer cells proved to proliferate significantly less. Results are represented as mean (SD) of 3 independent experiments. One-way ANOVA was employed to test for differences between groups and indicated as ** *p* ≤ 0.01, *** *p* ≤ 0.001, and **** *p* ≤ 0.0001 compared with controls.

To prove that PAFR upregulation significantly contributes to cancer cell proliferation, we reduced PAFR expression through siRNA interference in UWB1.289 cells. Three specific siRNA sequences significantly reduced PAFR expression compared to the negative control (*p* ≤ 0.0001; Figure 3a). Cell proliferation was confirmed to be significantly lower in the PAFR knockdown cancer cells than in the cells used as control group (*p* = 0.0003; Figure 3b).

### 3.5. Rupatadine Has an Inhibitory Effect on Proliferation and Migration of Ovarian Cancer Cells

**Figure 4:**
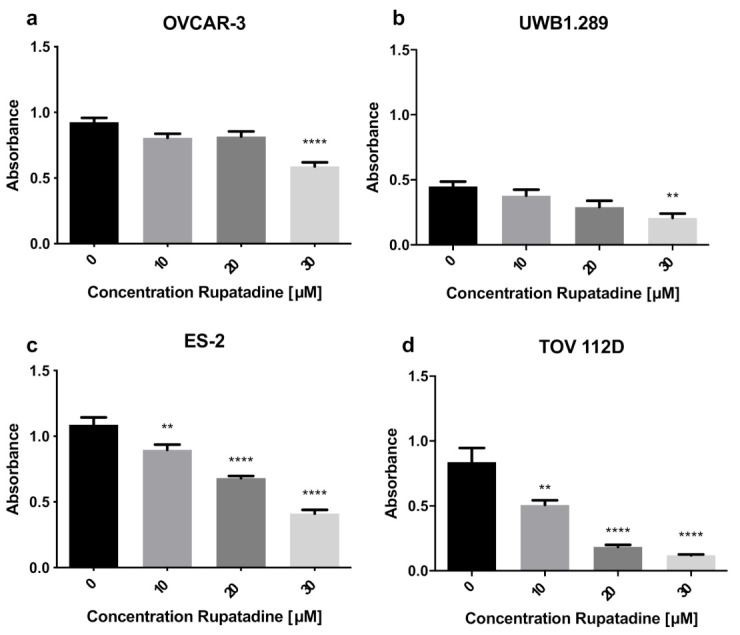
Rupatadine decreases proliferation of ovarian cancer cells. (**a**) OVCAR-3, (**b**) UWB1.289, (**c**) ES-2, and (**d**) TOV 112D were treated with increasing concentrations of rupatadine. Cell viability was tested with the MTT assay. All cell lines showed a concentration-dependent decrease in cell proliferation, with the greatest effect obtained with 30 μM of rupatadine ((**a**,**c**,**d**) *p* ≤ 0.0001, (**b**) *p* = 0.0021). In addition, 10 μM and 20 μM of rupatadine had a significant antiproliferative effect in (**c**) ES-2 cells (control vs. 10 μM of rupatadine, *p* = 0.0048; and 20 μM of rupatadine, *p* ≤ 0.0001) and (**d**) TOV 112D cells (control vs. 10 μM of rupatadine, *p* = 0.0016; and 20 μM of rupatadine, *p* ≤ 0.0001). Results are represented as mean (SD) of 3 independent experiments. One-way ANOVA was employed to test for differences between groups and indicated as ** *p* ≤ 0.01 and **** *p* ≤ 0.0001 compared with controls.

To investigate the effect of the PAFR antagonist rupatadine on ovarian cancer, we performed functional assays in different ovarian cancer cells. All cell lines demonstrated a significantly reduced cell viability after being treated with rupatadine. This effect was highlighted by the response of OVCAR-3, UWB1.289, ES-2, and TOV 112D cells to 30 μM of rupatadine (Figure 4). In particular, OVCAR-3, ES-2, and TOV 112D ovarian cancer cells responded to 30 μM rupatadine with a strong antiproliferative effect (*p* ≤ 0.0001). Lower concentrations of rupatadine (10 μM and 20 μM) diminished proliferation notably in endometrioid and clear cell ovarian cancer cells.

**Figure 5:**
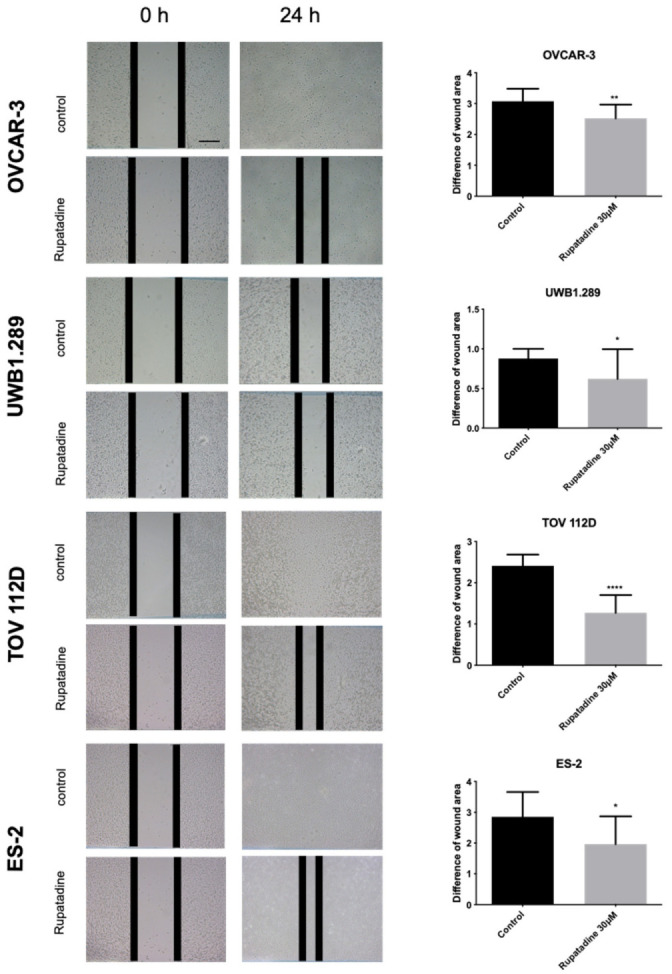
Treatment with rupatadine reduces cell migration of ovarian cancer cells. Wound area was measured at 0 h and 24 h in the control and 30 μM rupatadine group. Four different ovarian cancer cell lines showed significantly reduced migration after treatment compared to the control group (scale bar: 200 µm). Columns represent the measured difference of the area covered by cells between 0 h and 24 h. Results are represented as mean (SD) of 3 independent experiments. Unpaired *t*-test was employed to test for differences between groups and indicated as * *p* ≤ 0.05, ** *p* ≤ 0.01, and **** *p* ≤ 0.0001 compared with controls.

As a next step, we wanted to assess rupatadine’s influence on ovarian cancer cell migration. The wound healing assay was performed, showing significantly less cell migration in all cancer cell lines after 24 h compared to the control group (OVCAR-3: *p* = 0.0044, UWB1.289: *p* = 0.0261, TOV 112D: *p* ≤ 0.0001, and ES-2: *p* = 0.0145; Figure 5).

## 4. Discussion

The results of this study demonstrate the clinical importance of PAFR expression in ovarian cancer patients. Previously, it has been shown that PAFR is overexpressed in ovarian cancer samples [11]. To our knowledge, this is the first study evaluating the effect of PAFR expression on long-term ovarian cancer patients’ outcomes. In those patients, high cytoplasmic PAFR expression was significantly associated with worse overall survival and shorter recurrence-free survival time. Raised cytoplasmic PAFR was proved to be an independent prognostic factor by a multivariate Cox-analysis. 

Other studies have alluded to the differences of PAFR expression in distinct ovarian cancer subtypes [11,24]. In line with other studies, our data confirmed that the non-mucinous ovarian cancer patients displayed a significantly elevated PAFR staining intensity compared to the mucinous subtype. Patients with positive cytoplasmic PAFR expression demonstrated a significant correlation with histology, but more interestingly, with high grading in serous cancer cells. When comparing the various ovarian cancer cells in vitro, the serous and the BRCA1 mutated ovarian cancer cells significantly overexpressed PAFR. 

The breast cancer 1/2 (BRCA1/2) genes are important players in the pathogenesis of ovarian cancer [25,26]. They have several essential caretaker roles, such as transcriptional regulation and tumor suppressors in normal epithelial cells [27]. In 15–20% of ovarian cancer patients, a mutation in the tumor suppressor genes breast cancer 1/2 (BRCA1/2) can be found, leading to a familial accumulation of ovarian and breast cancer. BRCA1 mutation will increase the risk of developing ovarian cancer to about 40–60%, compared to the cumulative risk of about 1.3% in the general population [28]. 

In our study, we could not only show that the BRCA1 mutant cell line UWB1.289 displayed a higher PAFR expression than the rest of the epithelial OC cells but also that siRNA knockdown of PAFR in this cell line significantly decreased cancer cell proliferation. The role of PAF and its receptor in BRCA1-mutated ovarian cancer cells has also been examined in other studies. Zhang et al. have focused on the molecular pathways of PAF/PAFR and their association with early malignant transformation in BRCA1-mutated ovarian cells [24]. By the protein–protein interaction between PAFR and FAK and FAK and the Signal Transducers and Activators of Transcription (STAT1), the receptor promoted tumor progression and contributed to early malignant transformation of BRCA1-mutant ovarian epithelial cells. 

In general, the pro-inflammatory PAF/PAFR axis has been shown to play a crucial role in anti-apoptosis, tumor growth, and metastasis [29,30,31,32,33]. Beside tumor cells, cells of the tumor microenvironment such as monocytes/macrophages, polymorphonuclear leukocytes, platelets, and endothelial cells express PAFR. It has been shown that PAFR knockdown influences immune cell activation in the tumor microenvironment, thereby influencing tumor growth [34]. Further studies need to show the role of PAFR antagonism in the tumor microenvironment of ovarian cancer.

The receptor induces downstream signaling via EGFR/Src/FAK/Paxillin [11]. Known PAFR antagonists are, amongst others: WEB-2086, Ginkgolide B, and rupatadine [16,35,36,37]. Rupatadine is a PAFR antagonist that is clinically used as an antihistaminic drug [17,38]. Studies on rupatadine have primarily focused on its anti-inflammatory effects, such as the inhibition of mast cell degranulation and eosinophil chemotaxis [38]. This is the first study investigating rupatadine’s effect on cancer cells. We could demonstrate that rupatadine can effectively inhibit in vitro cell proliferation and migration of clear cell, serous, BRCA1 mutant, and endometrioid ovarian cancer cells. Hence, rupatadine seems to have a significant antiproliferative effect on ovarian cancer cells.

However, rupatadine is not only an antagonist of PAFR but also of the histamine H1 receptor [16]. Functional knockdown of the PAFR as shown in Figure 3 decreases UWB1.289 proliferation by half. BRCA1-mutated ovarian cancer cells (UWB1.289) significantly overexpressed PAFR. Thus, any further reduction of proliferation by rupatadine may be attributed to an antagonism of the histamine H1 receptor. It has been shown that endometrial cancer cells express elevated levels of H1 receptor [39]. This expression might be an explanation for an enhanced reduction of proliferation in endometrial cancer cells (TOV 112D), as seen in Figure 4b. To our knowledge, the role of histaminic receptors in ovarian cancer has not been explored so far and seems to be an interesting field of research for the future.

The effect of other PAFR antagonists, such as WEB-2086 and Ginkgolide B, on ovarian cancer cells has already been studied [14,24]. Compared to the two substances, rupatadine’s anti-PAF activity appears to be lower [16]. However, in contrast to WEB-2086, rupatadine is a drug that has already been tested in a multicenter phase-IV study and is clinically approved [16]. Several studies have confirmed rupatadine’s long-term safety profile [40]. Thus, using rupatadine as a PAFR-antagonist and thereby reducing tumor growth in ovarian cancer is a treatment option worth considering. The following studies will need to confirm rupatadine’s antiproliferative effect on ovarian cancer in vivo.

It is noteworthy that in melanoma and ovarian cancer cells, PAFR antagonists have been demonstrated to have a potentiating effect of chemotherapeutic drugs [37]. Yu et al. provided evidence that PAFR antagonists sensitized ovarian cancer cells to cisplatin, and the combined treatment reduced tumor growth [15]. Similarly, the combined PAFR and EGFR inhibition synergistically diminished ovarian cancer progression [14]. In further studies, the combined effect of rupatadine with other chemotherapeutic drugs could be studied in translational models, evaluating whether a combination can improve tumor therapy. Using retrospective data, it would be interesting to determine whether ovarian cancer patients who received rupatadine as an antihistaminic drug had a better outcome. To complement our data, additional analysis should focus on the prognostic relevance of PAFR expression in BRCA1 mutant ovarian cancer specimens on long-term survival.

## Figures and Tables

**Table 1:** Patient characteristics.

Parameters	N	Percentage
Histology		
serous	110	70.5%
clear cell	12	7.7%
endometrioid	21	13.5%
mucinous	13	8.3%
**Lymph Node**		
pNX	61	39.1%
pN0	43	27.6%
pN1	52	33.3%
**Distant Metastasis**		
pM0/X	150	96.2%
pM1	6	3.8%
**Grading**		
*serous*		
low	24	21.8%
high	80	72.7%
*endometrioid*		
G1	6	28.6%
G2	5	23.8%
G3	8	38.1%
*mucinous*		
G1	6	46.2%
G2	6	46.2%
G3	0	0%
*clear cell*		
G3	12	100%
**FIGO**		
I	35	22.4%
II	10	6.4%
III	103	66.0%
IV	3	1.9%
**Age**		
(median) years	62 ± 12	
Deaths	100	

**Table 2:** Correlation analysis between positive cytoplasmic PAFR expression and clinicopathological data.

Variables	*p*	Correlation Coefficient
**Histology**	0.011	−0.222
**FIGO**	0.749	0.029
**Grading**		
*serous-low grading*	0.647	0.040
*serous-high grading*	0.031	0.189
*clear cell, endometrioid* *and mucinous-G1 to G3*	0.051	−0.174

**Table 3:** Multivariate analysis of clinicopathological parameters and cytoplasmic PAFR expression.

Covariate	Coefficient (b_i_)	HR [Exp(b_i_)]	95% CI		*p*-Value
			Lower	Upper	
**Histology**	0.335	1.398	0.809	2.418	0.230
**FIGO** **(I, II vs. III, IV)**	1.462	4.316	2.222	8.384	0.000
**Grading**					
*serous low*	−0.526	0.591	0.186	1.883	0.374
*serous high*	0.692	1.999	0.742	5.386	0.171
*clear cell, endometrioid and mucinous-G1 to G3*	0.043	1.044	0.685	1.591	0.841
**Patients’ age (≤45 vs. >45 years)**	−0.196	0.822	0.416	1.624	0.572
**PAFR** **cytoplasmic** **IRS > 3**	1.403	4.069	1.031	16.059	0.045

## Data Availability

The datasets generated and/or analyzed during the current study are available from the corresponding author upon reasonable request.

## Supplementary Materials

The following are available online at https://www.mdpi.com/article/10.3390/cells10092337/s1, Figure S1: Expression of PAFR in different ovarian cancer cell lines, Table S1: Sequences of primers used in qPCR to determine mRNA expression levels, Table S2: Sequences of siRNA against PAFR mRNA, Table S3: Immunoreactive score of analyzed tissue microarrays.

## Abbreviations

BRCA	breast cancer gene
DNA	deoxyribonucleic acid
EOC	epithelial ovarian cancer
FBS	fetal bovine serum
FIGO	International Federation of Gynecology and Obstetrics
GAPDH	glycerinaldehyd-3-phosphat-dehydrogenase
IHC	immunohistochemistry
IRScore	immunoreactive score
mRNA	messenger ribonucleic acid
MTT	3-(4,5-dimethylthiazol-2-yl)-2,5-diphenyltetrazolium bromide
OS	overall survival
PAF	platelet-activating factor
PTAFR	platelet-activating factor receptor
qPCR	quantitative polymerase chain reaction
RFS	recurrence-free survival
ROC	receiver operating characteristic
siRNA	small interfering ribonucleic acid
SD	standard deviation
Src/FAK	steroid receptor coactivator/focal adhesion kinase
STAT	signal transducer and activator of transcription
