# Supplementary material for: The Platelet-Activating Factor Receptor’s Association with the Outcome of Ovarian Cancer Patients and Its Experimental Inhibition by Rupatadine
Source: Cells. 2021 Sep 7;10(9):2337. doi: 10.3390/cells10092337 (PMC8466210; doi:10.3390/cells10092337)
Supplement: Supplementary file 1 [file cells-10-02337-s001.zip › cells-1300857-supplementary.pdf]

Supplementary Materials

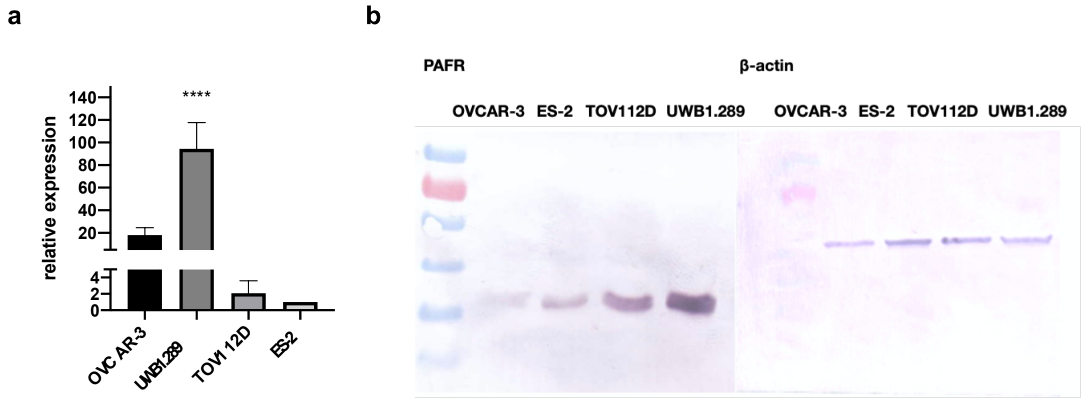

Figure S1. Expression of PAFR in different ovarian cancer cell lines. **(a)** PCR analysis of the four histological subtypes revealed an over-expression of the PAFR in serous cell lines (UWB1.289 and OVCAR-3). Results are represented as mean (SD) of 3 independent experiments. The difference was calculated relative to the ES-2 cell line with the ordinary one-way ANOVA test; $^{**}p = < 0.01$, $^{***}p = < 0.001$ and $^{****}p = < 0.0001$ compared with controls. **(b)** Especially the BRCA1 mutated cell lines showed an elevated protein expression.

Table S1. Sequences of primers used in qPCR to determine mRNA expression levels.

**PAFR**	Forward: GGGGACCCCCATCTGCCTCA<br>Reverse: GCGGGCAAAGACCCACAGCA
**β-actin**	Forward: TCCTCCCTGGAGAAGAGCTA<br>Reverse: CGTGGATGCCACAGGACT
**GAPDH**	Forward: AGCCACATCGCTCAGACAC<br>Reverse: GCCCAATACGACCAAATCC

Table S2. Sequences of siRNA against PAFR mRNA.

**S1**	CGCCTGTACCCTTGCAAGAAA
**S3**	ACCACGGATACGGTCACTGAA
**S5**	CACGGTCTTGGCGGTGTTCAT

Table S3. Immunoreactive score of analyzed tissue microarrays.

**Patient number**	**IRS of PTAFR**
1	4,00
2	4,00
3	6,00
4	4,00
5	8,00
6	4,00
7	4,00
8	8,00
9	8,00
10	4,00
11	4,00
12	4,00
13	4,00
14	8,00
15	8,00

16	8,00
17	8,00
18	8,00
19	4,00
20	4,00
21	4,00
22	8,00
23	4,00
24	6,00
25	6,00
26	8,00
27	4,00
28	4,00
29	6,00
30	6,00
31	8,00
32	6,00
33	8,00
34	8,00
35	4,00
36	8,00
37	4,00
38	8,00
39	4,00
40	8,00
41	8,00
42	8,00
43	6,00
44	4,00
45	3,00
46	4,00
47	8,00
48	8,00
49	4,00
50	4,00
51	8,00
52	4,00
53	8,00
54	4,00
55	8,00
56	4,00
57	8,00
58	8,00
59	4,00
60	8,00
61	8,00
62	8,00
63	8,00
64	4,00
65	3,00
66	4,00
67	8,00
68	4,00
69	6,00
70	4,00
71	4,00
72	8,00
73	8,00

74	4,00
75	8,00
76	8,00
77	8,00
78	8,00
79	4,00
80	8,00
81	8,00
82	8,00
83	4,00
84	8,00
85	8,00
86	8,00
87	8,00
88	4,00
89	8,00
90	8,00
91	12,00
92	4,00
93	4,00
94	4,00
95	4,00
96	6,00
97	12,00
98	4,00
99	8,00
100	4,00
101	8,00
102	4,00
103	8,00
104	4,00
105	4,00
106	8,00
107	4,00
108	8,00
109	8,00
110	6,00
111	8,00
112	8,00
113	6,00
114	4,00
115	8,00
116	8,00
117	2,00
118	8,00
119	8,00
120	9,00
121	4,00
122	4,00
123	4,00
124	8,00
125	6,00
126	4,00
127	3,00
128	4,00
129	4,00
130	8,00
131	2,00

132	4,00
133	8,00
134	4,00
135	4,00
136	8,00
137	2,00
138	2,00
139	6,00
140	6,00
141	1,00
142	1,00
143	3,00
144	2,00
145	0,00
146	2,00
147	2,00
